# Stereotactic radiotherapy on brain metastases with recent hemorrhagic signal: STEREO-HBM, a two-step phase 2 trial

**DOI:** 10.1186/s12885-020-6569-1

**Published:** 2020-02-22

**Authors:** Paul Lesueur, William Kao, Alexandra Leconte, Julien Geffrelot, Justine Lequesne, Joëlle Lacroix, Pierre-Emmanuel Brachet, Ioana Hrab, Philippe Royer, Bénédicte Clarisse, Dinu Stefan

**Affiliations:** 1 Radiation Oncology Department, Centre François Baclesse, F-14000 Caen, France; 2 Normandy University, F-14000 Caen, France; 3 Clinical Research Department, Centre François Baclesse, F-14000 Caen, France; 4 Radiology Department, Centre François Baclesse, F-14000 Caen, France; 5 Medical Oncology Department, Centre François Baclesse, F-14000 Caen, France; 6 Radiation Oncology Department, Institut de Cancérologie de Lorraine, F-54000 Vandœuvre-lès-Nancy, France; 7 Radiation Oncology Department, Centre François Baclesse, 3 Avenue du Général Harris, F-14076 Caen Cedex 05, France

**Keywords:** Stereotactic radiotherapy, Brain metastases, Bleeding, Quality of life

## Abstract

**Background:**

Brain metastases often occur in cancer evolution. They are not only responsible for death but also for disorders affecting the quality of life and the cognitive functions.

Management of brain metastases usually consists in multi-modality treatments, including neurosurgery, whole brain radiotherapy (WBRT), and more recently radiosurgery (SRS) or fractionated stereotactic radiotherapy (FSRT), systemic treatment (chemotherapy or targeted therapy), combined or not with corticosteroids. Almost 20% of brain metastases can present recent (within 15 days) bleeding signs on neuro-imagery. In these conditions, WBRT is the usual treatment. Yet, patients may benefit from a more aggressive strategy with SRT or FSRT. However, these options were suspected to possibly major the risk of brain haemorrhage, although no scientifically proven. Radiation oncologists therefore usually remain reluctant to deliver SRS/FSRT for bleeding brain metastases.

It is therefore challenging to establish a standard of care for the treatment of bleeding brain metastases.

We propose a phase II trial to simultaneously assess safety and efficacy of FSRT to manage brain metastases with hemorrhagic signal.

**Methods:**

The STEREO-HBM study is a multicenter two-step non-randomised phase II trial addressing patients with at least one bleeding brain metastasis out of a maximum of 3 brain metastases. Each brain metastasis will be treated with 30 Gy in 3 fractions for 1 week.

The main endpoint is based on both safety and efficacy endpoints as proposed by Bryant and Day’s design. Safety endpoint is defined as the rate of bleeding complications 4 months post-FSRT while efficacy endpoint is defined as the 6-month local control rate. Multi-modal MRI will be used to assess intra-tumoral hemorrhagic events before and after treatment. Patients’ quality of life will also be assessed.

**Discussion:**

Management of bleeding brain metastases is still debated and poorly explored in clinical trials. There is sparse and weak data on the signification of pretreatment intra-tumour haemorrhagic signs or on the risk of brain bleeding complications after FSRT.

We expect this first prospective phase 2 trial in this particular setting will allow to clarify the place of FSRT to optimally manage bleeding brain metastases.

**Trial registration:**

NCT 03696680, registered October, 4, 2018.

**Protocol version:**

Version 2.1 dated from 2018/11/09.

## Background

Brain metastases occur in 20–40% of cancer patients. They represent the most common manifestation of intracranial malignancy [1]. They are an important cause of mortality and morbidity. Indeed, brain metastases can result in devastating clinical consequences, such as sensitive-motor defect, cognitive disturbance, social relationship deterioration. Without any specific treatment, patients with brain metastases usually survive for 1 to 2 months [2, 3]. For these patients with brain evolution of their cancer, death results from the extra-cerebral disease progression in most of cases, but from complications related to brain lesions progression in at least 25–50% of cases [4, 5].

Brain metastases exhibit highly variable revelations modes. They can be asymptomatic or otherwise occur more abruptly. An epileptic seizure or loss of consciousness may reveal brain damage. In that latter case, it is estimated that 1.9 to 10% of these symptoms are associated with intra-tumoral haemorrhage [6]. Bleeding risk varies depending on histology. For example, melanoma metastases are macroscopically bleeding in 35.7% of cases, whereas 2.9 and 4.7% of metastases from adenocarcinoma or anaplastic carcinoma are bleeding, respectively [7]. Overall, almost 20% of brain metastases can present recent (within 15 days) bleeding signs on neuro-imaging (Magnetic Resonance Imaging (MRI) or Scan).

Although radiosurgery (SRS) or fractionated stereotactic radiotherapy (FSRT) is now the mainstay of treatment for brain oligo-metastases (3–5 metastases), allowing a 12-month local control greater than 75% [8], whole brain radiotherapy (WBRT) still remains the usual treatment of haemorrhagic brain metastases, despite its poor efficacy, namely a 6-month and 12-month local control rate of 37 and 15%, respectively [9]. This attitude is consistent with the report of the French High Authority of Health (HAS) which does not support radiosurgery for the treatment of haemorrhagic brain metastases (HAS report 2001). It is based on the results from a retrospective study (131 metastases on 54 patients) [10]: haemorrhage was identified in 7.4% of the metastases before radiosurgery and in 18.5% of the metastases after radiosurgery. Since this publication, although it did not clearly demonstrate a relationship between radiosurgery and the risk of haemorrhage, FSRT/SRS is suspected to increase the risk of brain haemorrhage. Furthermore, in spite of several reports of intra-tumor haemorrhage after radiosurgery of brain metastases, radiosurgery was not shown to increase the incidence of haemorrhage. Thus, among melanoma patients carrying brain metastases [11], the rate of intra-tumor haemorrhage was shown to be similar before and after treatment by stereotactic Gammaknife (23.7% vs. 15.2%, *p* = 0.89); the presence of intra-tumoral bleeding before treatment was not found to major the risk of bleeding after treatment (*p* = 0.9). According to some authors, the occurrence of post-treatment bleeding would not be related to the achievement of radiosurgery, but rather to the intrinsic sensitivity of the tumor to bleed [12].

Besides these conflicting findings, it has to be highlighted that most of these studies were conducted exclusively with SRS (a single fraction issued) and from either a Gammaknife® or a linear adapted accelerator. To date, there are no specific available data for FSRT (several fractions) with Cyberknife®, a newer technology.

Overall, radiation oncologists generally remain reluctant to deliver FSRT on hemorrhagic brain metastases. Therefore, the standard treatment remains panencephalic irradiation, even if it is clearly not optimal.

In this context, there is a real need to establish a standard management of hemorrhagic brain metastases, notably using more innovative radiotherapy techniques like FSRT.

In order to specifically document the interest of FSRT in the management of hemorrhagic brain metastases, we propose the first non-randomized phase 2 prospective trial aiming to simultaneously evaluate safety and efficacy of this treatment. In addition, it will accurately document, using multi-modal MRI, intra-tumoral hemorrhagic events before and after treatment. Patients’ quality of life before and after treatment will be also assessed.

## Methods/design

### Trial objectives

#### Primary objective

The main objective is based on joint primary endpoints of safety and efficacy of FSRT for patients with bleeding brain metastases at diagnosis, as proposed by the Bryant-and-Day design [13].

The safety endpoint is the rate of hemorrhagic complications (MRI signal modifications with or without clinical manifestation) occurring within 4 months after the end of FSRT [14, 15], defined as the proportion of patients with at least one target brain metastasis with a bleeding complication within 4 months post-FSRT.

The efficacy endpoint is the local control rate of irradiated target lesions (all irradiated brain lesions with stable size or size increase less than 25%) 6 months after the end of FSRT, using RECIST 1.1 criteria.

Targets lesions correspond to all irradiated lesion regardless the presence of a bleeding signal.

#### Secondary objectives

The secondary objectives are to evaluate:

- safety profile (all acute and late toxicities according to EORTC criteria)
- intra-cerebral progression-free survival (excluding irradiated lesions)
- extra-cerebral progression-free survival
- overall survival
- quality of life evolution at short, mid and long term using EORTC QLQ-C30 and QLQ-BN20 questionnaires
- survival without any toxicity (grade ≥ 2) including quality of life (QoL) impairment (of ≥10 points out of a 100-point scale in at least one dimension of QoL), nor tumor progression (Q-TWIST)
- the prevalence of modifications after FSRT on morphological, functional and spectro-MRI parameters

### Study population

Eligibility criteria are detailed in Table 1. More specifically, the targeted patients had to carry up to 3 brain metastases of solid tumor [16, 17], measuring 5–30 mm in diameter, eligible to stereotactic radiotherapy, of which at least one lesion presented signs of intra-tumor bleeding [18] before stereotactic irradiation.

**Table 1:** Study eligibility criteria

Inclusion criteria	- Age > 18 years old - WHO performance status 0 or 1 - Patient having less than 4 brain metastases of solid tumour with a histologically proven diagnosis of solid tumour; patients who have had a metastasectomy and having 1 to 3 brain metastases are eligible; - Brain(s) lesion(s) measuring between 5 and 30 mm in diameter - Patient eligible for stereotactic radiotherapy after a local multidisciplinary committee decision - Signs of intra-tumour bleeding before stereotactic irradiation in at least one brain metastasis and defined on the presence of at least one of these criteria: • Spontaneous high-density lesion on brain CT scan without injection • Spontaneous hyper-intense lesion on brain MRI sequences: on T1 sequence • Lesion with hypo signal on T2* sequences - Patients with an extra-cranial control disease treated with systemic therapy (chemotherapy, immunotherapy or targeted therapy) could be included only if they show a: • complete response disease • partial response or stable disease for more than 3 months - Patient sufficiently cooperating to perform the treatment with the use of a thermoformed mask; - Patient whose neuropsychological abilities allow to follow the requirements of the protocol; - Signed informed consent.
Exclusion criteria	- Patients with small cell lung cancer, germ-cell tumors, lymphoma, melanoma, leukemia and multiple myeloma are not eligible; - Patients with an associated neurodegenerative disease; - Any symptoms not attributable to brain metastasis or cancer disease requiring long term corticosteroid use (regardless of dose); - Contraindication to perform the brain MRI, or to infuse gadolinium or iodinated contrast product - Bleeding disorders; - Genetic disorder leading to hyper radiosensitivity (Neurofibromatosis, ataxia-telangiectasia ...); - Thrombocytopenia < 100,000 cells / mm3; - Anticoagulant therapy with curative intent dosing (deep vein thrombosis …), and/or anti-platelet aggregation during FSRT - Hemorrhagic metastasis of the brainstem; - Patients for whom a treatment plan dedicated to one of the metastasis delivers more than 5 Gy on the other brain metastasis; - Patients with previous brain stereotactic irradiation - Whole brain irradiation history; - Progressive extracranial disease; - Any geographical conditions, social and associated psychopathology that may compromise the patient’s ability to participate in the study; - Participation in a therapeutic trial for less than 30 days; - Patient deprived of liberty or under guardianship.

### Trial design

The study protocol and this manuscript have been written in accordance with standard protocol items, namely recommendations for interventional trials (SPIRIT).

**Fig. 1:**
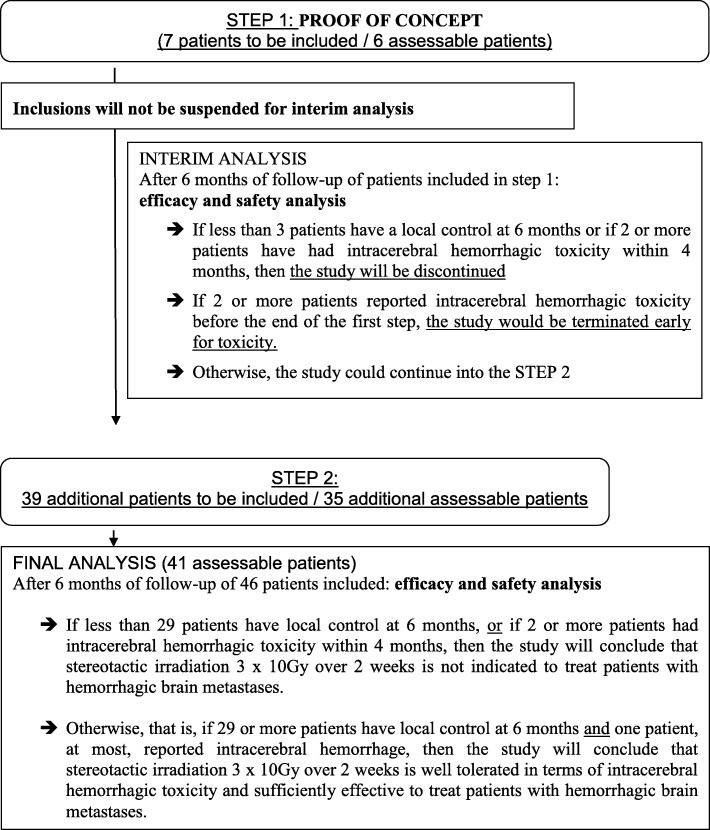
Methodology design of the STEREO-HBM study

The STEREO-HBM study is a multicenter 2-step non-randomised phase II trial where 46 patients are planned to be enrolled (Fig. 1). The study is based on both tolerance and clinical efficacy as proposed by Bryant and Day’s which allows simultaneous evaluation of clinical response and toxicity [13].

### Study sites

The list of study sites is available on https://clinicaltrials.gov/ct2/show/NCT03696680.

### Study treatment

Each targeted brain metastasis (hemorrhagic or not) will be treated at the dose of 30 Gy in 3 fractions at 10 Gy/fraction every 2 days [19, 20]. All target lesions (maximum 3 brain metastases plus one tumor bed) will be treated as much as possible over 1 week. However, cerebral irradiation of all the lesions may be spread over 7–10 calendar days. The irradiation facility could be LINAC (Truebeam STX®, Versa HD®, Novalis® …) or robotic radiosurgery system (Cyberknife®).

A minimum of 95% of the target volume (PTV) should receive at least 95% of the total prescribed dose of 30Gy (V95 > 28.5Gy).

The target volumes will be defined as [21, 22]:

- GTV (Gross tumor volume): Gadolinium enhanced volume or surgical tumor bed
- CTV (clinical target volume) = [GTV + 1 mm]
- SM (set-up margins) = 1–2 mm according to the technique or irradiation system used
- PTV (planning target volumes) = CTV + SM

Organ at risk will be delineated according to investigator habits (Optic chiasm, Optic nerves, Brainstem, Cochlea, Spinal Cord, Eyes). The prescription isodose percentage should be higher than 70%.

### Study procedures

**Fig. 2:**
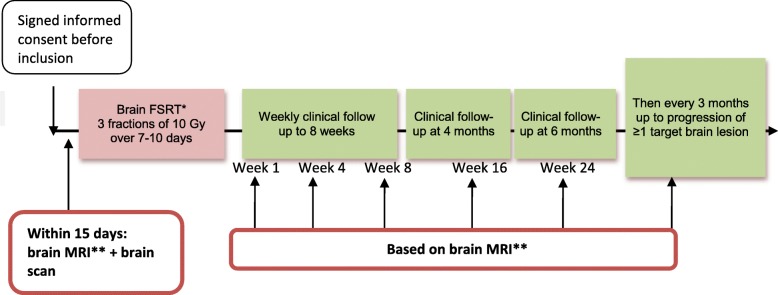
Schematic representation of the STEREO-HBM study. *Each targeted brain metastasis (hemorrhagic or not) will be treated at the dose of 30 Gy in 3 fractions at 10 Gy per fraction every 2 days. All target lesions (maximum 3 brain metastases plus one tumor bed) will be treated as much as possible over 1 week. However, cerebral irradiation of all the lesions may be spread over 7–10 calendar days. **Standard MRI imaging protocol plus optional multivoxel spectroscopy imaging (MSI) only for voluntary patients with specific signed informed consent. Abbreviation: FSRT hypofractionated stereotactic radiotherapy; MRI Magnetic Resonance Imaging. *Each targeted brain metastasis (hemorrhagic or not) will be treated at the dose of 30 Gy in 3 fractions at 10 Gy per fraction every 2 days. All target lesions (maximum 3 brain metastases plus one tumor bed) will be treated as much as possible over 1 week. However, cerebral irradiation of all the lesions may be spread over 7–10 calendar days. **Standard MRI imaging protocol plus optional multivoxel spectroscopy imaging (MSI) only for voluntary patients with specific signed informed consent. Abbreviation: FSRT hypofractionated stereotactic radiotherapy; MRI Magnetic Resonance Imaging

The trial schema is illustrated in Fig. 2. The overview of study assessments and procedures are detailed in Table 2.

**Table 2:** Table caption

	Before initiation of treatment D-15 to D0	Stereotactic irradiation week FSRT Treatment of all lesions in 7-10 days maximum	End of irradiation W1 (1 week after the end of irradiation)	Follow-up visits after treatment				Follow-up after progression^f^
				Each week up to 2 months after end of treatment W2 to W9	4 months after end of treatment W16	6 months after end of treatment W24	Every 3 months up to disease progression^c^	
Stereotactic irradiation		Each brain metastasis (hemorraghic or not) will be treated at 30 Gy in 3 fractions of 10 Gy / fraction every 2 days on maximum 7 days						
Signature of informed consent	✓							
Clinical exam including:								
- Disease medical history, weight, height, SC, PS) Patient pronostic (DS-GPA)	✓							
- Complete clinical evaluation and neurological examination		✓	✓	✓	✓	✓	✓	
- Evaluation of toxicities		✓(once a week)	✓	✓	✓	✓	✓	✓
Biological assessment^a^								
- Complete Blood Count	✓		✓	✓	✓	✓	✓	
- Creatinin-^**a**^	✓		✓	✓^b^	✓	✓	✓	
Imagery including:	✓							
- Cerebral scan	✓		✓	✓^b^	✓	✓		
- Cerebral MRI with T1 sequency Followed by Spectro-MRIe (ancillary study)	✓	✓ (in case of neurological degradation due to treated lesions)	✓	✓^b^	✓	✓	✓ ✓	
Quality of life questionnaires (EORTC QLQ C30 and BN20)	✓			✓ Only at 1 month	✓	✓	✓^d^	

^a^Creatinin must be performed before MRI

^b^4 weeks and 8 weeks after the end of irradiation (W4 and W8)

^c^Every 3 months up to at least one target irradiated lesion in disease progression

^d^only every 6 months

^e^Spectro-MRI is performed at the same time of standard cerebral MRI and only applies to patients who have given their signed consent

^f^After progression, a survival status will be collected every 3 months with persistent toxicities due to radiotherapy

#### Brain tumor evaluation

Brain tumoral evaluation will be in line with international guidelines [23]. It will be based on a brain MRI performed at baseline (before FSRT), at 1 week, 4 weeks, 8 weeks after the end of FSRT and thereafter at 4 months, 6 months and every 3 months post-FSRT in the absence of tumoral progression.

Each brain MRI will include the following sequences [19, 24, 25]: T1, T2, T2*, T1 with gadolinium and T2 FLAIR, and, if possible, MRI SWI (susceptibility-weighted imaging).

Disease assessment evaluation will be determined locally according to RECIST version 1.1 criteria.

#### Multi-modality MRI ancillary study

In addition to the standard MRI imaging protocol, each MRI imaging evaluation will include an optional multivoxel spectroscopy imaging (MSI) that will be performed only for voluntary patients with specific signed informed consent.

Perfusion and diffusion sequences will be added [26–28]. Evaluations may be helpful to explore the biochemistry of the tumor. Indeed, it appears important to be able to differentiate a tumor relapse from a therapeutic effect (radionecrosis) in the setting of this FSRT.

#### Quality of life assessment

Each patient will be asked to fil in standardized and validated self-administered questionnaires (EORTC QLQ-C30 and its specific brain cancer module BN-20) to assess health-related quality of life (QoL). QoL will be assessed at baseline, 4 weeks after the end of FSRT, thereafter 4 months, 6 months and every 3 months if no disease progression has occurred.

### Concomitant treatments

Authorized concomitant treatments include bisphosphonates and corticotherapy, prescribed at the discretion of the investigator, according to local practices.

The following treatments are prohibited:

- Systemic anticancer drugs (including chemotherapy, hormonotherapy, anti-angiogenics) have to be suspended at least 7 days prior to FSRT initiation and may be reintroduced 7 days after the last fraction.
- Anticoagulant drugs taken in a curative intent and platelet anti-aggregants have to be suspended at least 5 days prior to FSRT initiation and may be reintroduced 2 months after the end of FSRT

### Statistical design overview

The study will be conducted in 2 steps (a ‘proof of concept’ step followed by a ‘validation’ step) with a two-stage phase 2 design proposed by Bryant and Day [13], combining both safety and efficacy as primary endpoint (Fig. 1).

We posited the following assumptions:

- π_T0_ **≥** 0.15 and π_T1_ **≤** 0.05, the unacceptable and expected rate of hemorrhagic complications occurring within 4 months after the end of FSRT, respectively
- π_R0_ **≤** 60% and π_R1_ **≥** 80%, the unacceptable and expected local control rate of irradiated target lesions at 6 months, respectively.

With an alpha risk of 10% for both the efficacy and the toxicity, and a power of 90%, a total of 41 assessable patients are required.

The continuation of the study will depend on the results of the interim analysis.

Interim analysis will be performed after the first step: 6 assessable patients will be analyzed. Inclusions will not be suspended during the interim analysis. If less than 3 patients are locally controlled at 6 months or if 2 or more patients have presented an intracerebral hemorrhagic toxicity within 4 months, then the study will be discontinued for futility. If 2 or more patients reported intracerebral hemorrhagic toxicity before the end of the first step, the study would be terminated early for excess of toxicity. Otherwise, the study could continue into the second step: 35 additional assessable patients will be needed.

Final analysis will be performed after the second step. After a 6-month follow-up of the 41 assessable patients, if less than 29 patients are locally controlled at 6 months, or if 2 or more patients had intracerebral hemorrhagic toxicity within the 4 months following FSRT, then the study will conclude that FSRT (3 x 10Gy over 1 week) is not indicated to treat patients with hemorrhagic brain metastases. Otherwise, that is, if 29 or more patients are locally controlled at 6 months and if 1 patient, at most, reported intracerebral hemorrhage within 4 months post-FSRT, then the study will conclude that FSRT is effective, well tolerated and does not increase intracerebral hemorrhagic toxicity in patients with bleeding brain metastases.

Considering a drop-out rate of 10% (lost to follow-up, protocol deviation, etc.), 7 and 39 patients will be enrolled in the first and second step, respectively, for a total of 46 patients.

### Data management

A Web Based Data Capture (WBDC) system will be used for data collection and query handling. The investigator will ensure that data are recorded on the eCRFs as specified in the study protocol and in accordance with the instructions provided.

The investigator ensures the accuracy, completeness, and timeliness of the data recorded and of the provision of answers to data queries according to the Clinical Study Agreement. The investigator will sign the completed eCRFs. A copy of the completed eCRFs will be archived at the study site.

### Data monitoring committee

An Independent Data Monitoring Committee (IDMC) will be set-up to ensure the protection of patients, the ethical conduct of the study, to evaluate the benefit/risk ratio of the study, and to insure an independent review of the scientific outcomes during and at completion of the study. The IDMC exercises a consultative role for the promoter who takes the final decision for implementing the recommendations proposed by the IDMC. The committee will include a radiotherapist, an oncologist, a statistician and a pharmacologist.

### Withdrawal from study

Reasons for why a patient may discontinue participating to the study include:

- Patient request (withdrawal of consent for further treatment)
- Intolerable toxicity
- Concomitant disease or other reason requiring the discontinuation of treatment
- Patient lost to follow-up
- Investigator’s request (with detailed documentation of reasoning)

## Discussion

The scientific data studying the relationship between hypofractionated stereotactic radiotherapy (FSRT) or radiosurgery (SRS) for the management of hemorrhagic brain metastases, and the risk of intra-tumor and/or cerebral hemorrhage at the end of treatment are very insufficient, or contradictory.

In this context, we aim at assessing the interest of FSRT by proposing the first prospective phase 2 trial focusing on both safety and efficacy of this strategy for patients with bleeding brain metastasis.

In addition, intra-tumoral hemorrhagic events before and after treatment will be precisely documented, using multi-modal MRI. Patients’ health-related quality of life before and after treatment will be also assessed, using standardized validated self-administered questionnaires.

This project comes within a large scientific program of our Institution that aims at assessing various treatment approaches in primary and secondary brain tumours [29].

In the future, we hope the results of our prospective trial will reinforce that patients with hemorrhagic brain metastases could benefit from adapted and innovated treatment like FSRT, for optimal and safe management allowing maintaining quality of life.

## Data Availability

Not applicable.

## Abbreviations

ANOCEF: Association des Neuro-Oncologues d’expression française/Association of the neuro-oncologists of French expression
CTV: Clinical target volume
EORTC: European Organisation for Research and Treatment of Cancer
FSRT: Stereotactic radiotherapy
GTV: Gross tumor volume
HAS: French High Authority of Health
IDMC: Independent Data Monitoring Committee
MRI: Magnetic Resonance Imaging
MSI: Multivoxel spectroscopy imaging
PTV: Planning target volumes
QoL: Quality of Life
SM: Set-up margins
SPIRIT: Standard protocol items, namely recommendations for interventional trials
SRS: Radiosurgery
WBDC: Web Based Data Capture
WBRT: Whole Brain radiotherapy
WHO: World Health Organization
